# Supplementary material for: Probing Affinity, Avidity, Anticooperativity, and Competition in Antibody and Receptor Binding to the SARS-CoV-2 Spike by Single Particle Mass Analyses
Source: ACS Cent Sci. 2021 Nov 4;7(11):1863–73. doi: 10.1021/acscentsci.1c00804 (PMC8577368; doi:10.1021/acscentsci.1c00804)
Supplement: Supplementary file 1 — oc1c00804_si_001.pdf [file oc1c00804_si_001.pdf]

Probing Affinity, Avidity, Anti-Cooperativity, and Competition in Antibody and Receptor Binding to the SARS-CoV-2 Spike by Single Particle Mass Analyses

Victor Yin[1,2,*], Szu-Hsueh Lai[1,2,*], Tom G. Caniels[3], Philip J.M. Brouwer[3], Mitch Brinkkemper[3], Yoann Aldon[3], Hejun Liu[4], Meng Yuan[4], Ian A. Wilson[4,5], Rogier W. Sanders[3,6], Marit J. van Gils[3], Albert J.R. Heck[1,2,#]

[1]Biomolecular Mass Spectrometry and Proteomics, Bijvoet Center for Biomolecular Research and Utrecht Institute for Pharmaceutical Sciences, Utrecht University, Padualaan 8, 3584 CH, Utrecht, The Netherlands
[2]Netherlands Proteomics Center, Padualaan 8, 3584 CH, Utrecht, The Netherlands
[3]Department of Medical Microbiology and Infection Prevention, Amsterdam University Medical Centers, Location AMC, University of Amsterdam, Meibergdreef 9, 1105 AZ Amsterdam, The Netherlands
[4]Department of Integrative Structural and Computational Biology, The Scripps Research Institute, La Jolla, CA 92037, USA
[5]Skaggs Institute for Chemical Biology, The Scripps Research Institute, La Jolla, CA 92037, USA
[6]Department of Microbiology and Immunology, Weill Medical College of Cornell University, 1300 York Avenue, New York, NY 10065, USA

Contents:

Supplemental Figures S1 – S6

Supplemental Tables S1 – S4

Supplemental Figures

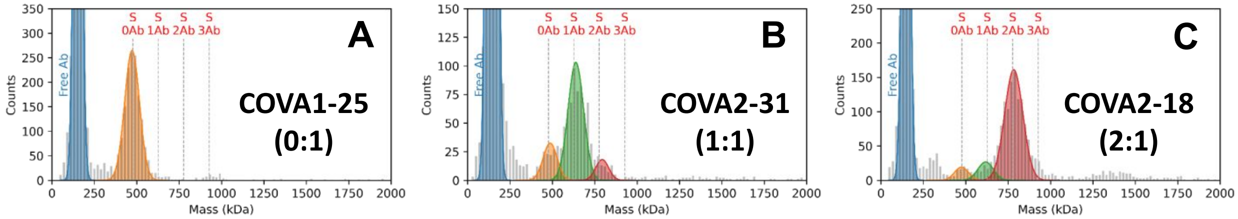

Figure S1. Representative MP histograms of three tested Abs (IgG format) bound to the S-trimer. (A) COVA1-25 (0:1 stoichiometry). (B) COVA2-31 (1:1 stoichiometry). (C) COVA2-18 (2:1 stoichiometry). As demonstrated here, different anti-S-trimer Abs can exhibit a variety of binding stoichiometries.

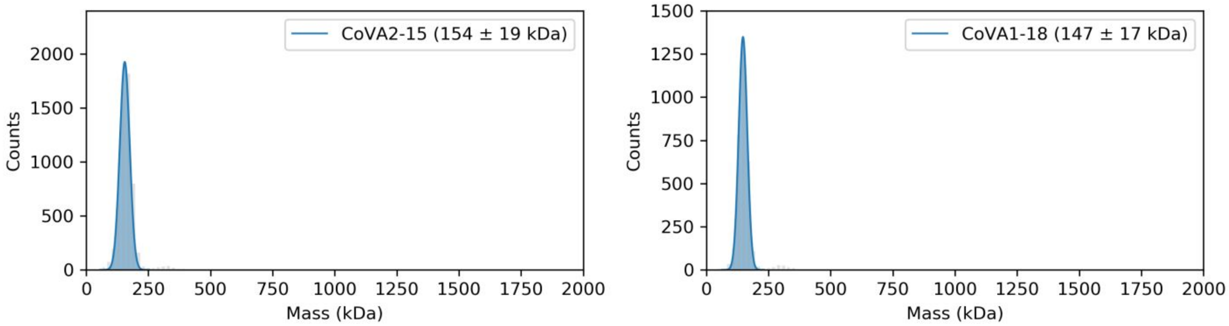

Figure S2. MP histograms of the COVA2-15 and COVA1-18 IgGs. A single mass distribution at ~150 kDa is observed for both Abs in line with the predicted masses.

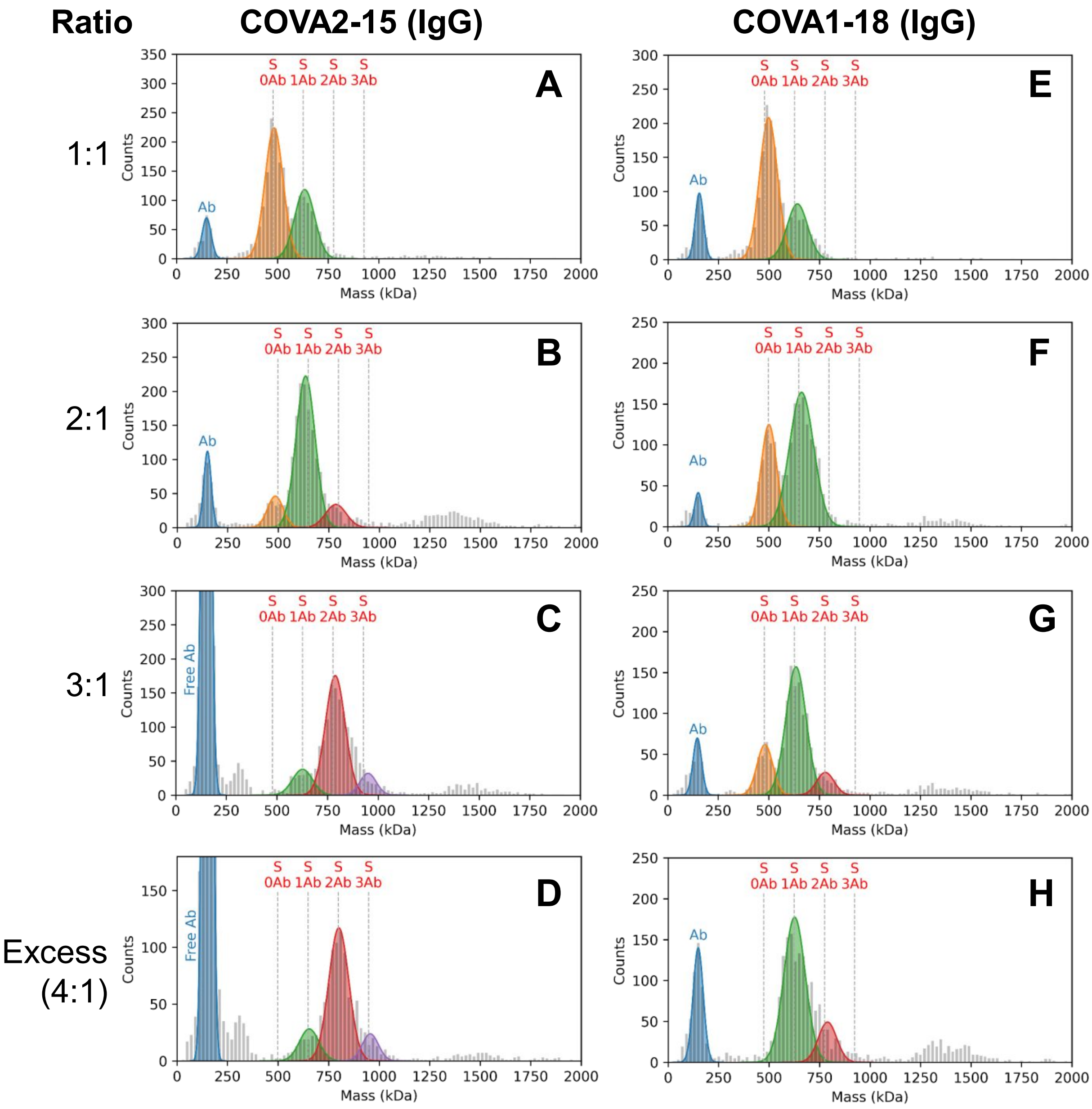

Figure S3. Stoichiometry of COVA2-15 and COVA1-18 binding to the S-trimer assessed by MP. MP histograms of COVA2-15 and COVA1-18 IgG binding to the SARS-CoV-2 S-trimer at different mixing ratios. (A-D): COVA2-15 Ab. (E-H): COVA1-18 Ab. The vertical dashed lines indicate the theoretical peak positions of each Ab-bound species. The data clearly reveal that full stoichiometric binding is not achieved for either Ab, but also that COVA2-15 preferably binds two Abs, whereas even at excess preferably just one COVA1-18 binds to the S-trimer. As expected, lower mixing ratios result in lower observed binding stoichiometries. The low-abundance signals observed between 1200 and 1600 kDa originate from Ab-binding induced S-trimer dimers. The measured masses and abundances related to these data are provided in **Supplemental Table S4**.

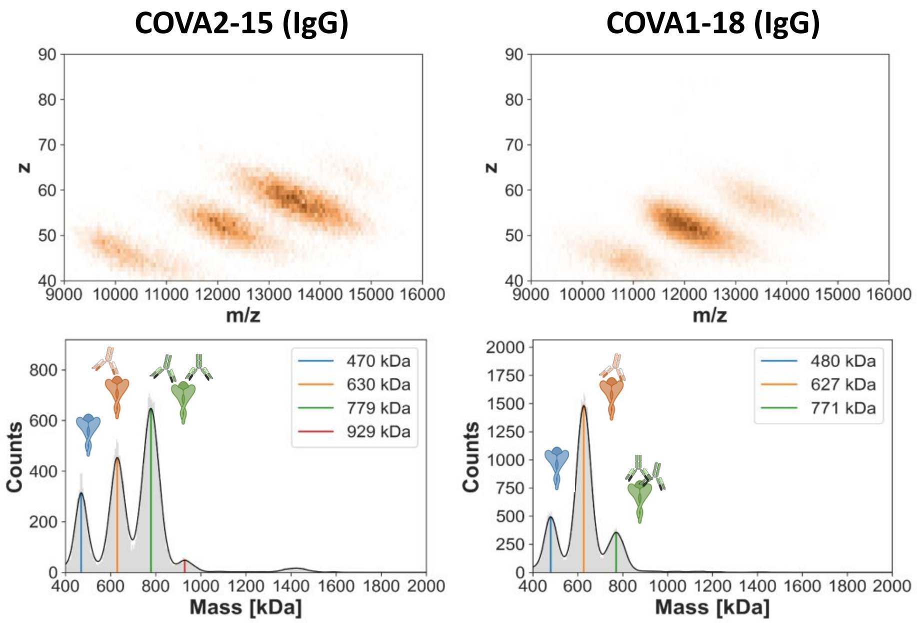

Figure S4. Stoichiometry of Ab binding to the S-trimer assessed by Orbitrap-based charge detection native mass spectrometry. Two-dimensional mass spectra of COVA2-15 and COVA1-18 Ab binding to the SARS-CoV-2 S-trimer at a mixing ratio of 4:1. Left: COVA2-15 Ab. Right: COVA1-18 Ab. The data are in line with the MP data presented in **Figure 2**, and also reveal that full stoichiometric binding is not achieved for either Ab, but also that COVA2-15 binds more readily two Abs, whereas even at access preferably just one COVA1-18 binds to the S-trimer. The measured masses and abundances related to these data and provided in **Supplemental Table S4**.

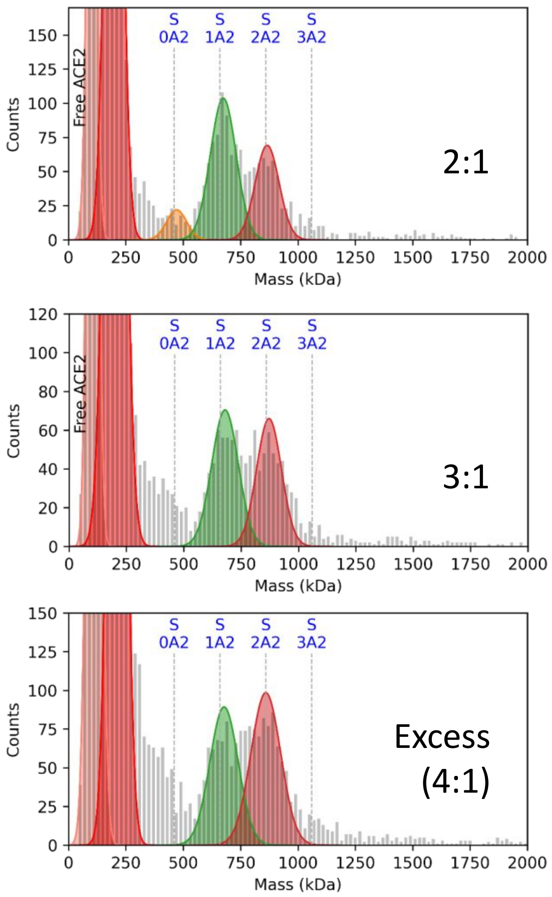

Figure S5. MP histograms of ACE2 binding to the S-trimer at increasing ratios. Even at an excess of 4:1, a substantial number of S-trimers still only bind one ACE2, and essentially no binding of 3 ACE2 is observed. Interference from a tetrameric ACE2 sub-population occlude accurate quantitation of the free S-trimer.

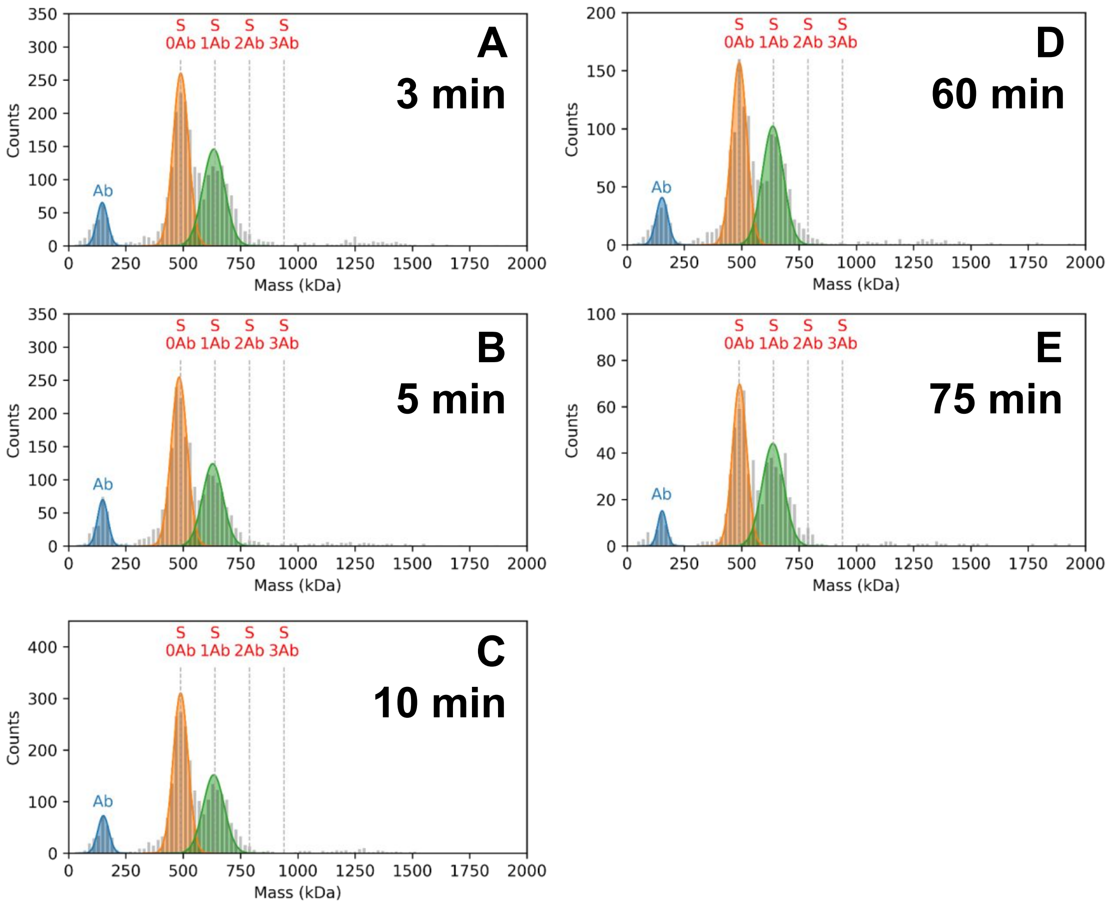

Figure S6. MP histograms of COVA2-15 binding to the S-trimer (1:1 ratio) at different incubation times. No major differences in binding populations are observed up to 75 minutes, confirming that samples are adequately equilibrated at the time of measurement. An incubation time of 5 minutes was used for all other measurements.

Supplemental Table S1. Tabulation of masses of SARS-CoV-2 S-trimer.

Method	Species	Mass (kDa)	FWHM (kDa)	Normalized Abundance (%)
MP	S (monomer)	139	73	8
MP	S (trimer)	475	106	92
CD-MS	S (monomer)	159	29	5
CD-MS	S (trimer)	477	61	95

Supplemental Table S2. Tabulation of fractional occupancies of each Ab bound to the S-trimer. Tabulated values are derived from the normalized summation of two 120 second MP acquisitions.

IgG	Fractional Occupancies			
	+0 Ab	+1 Ab	+2 Ab	+3 Ab
COVA1-25	1.000	0.000	0.000	0.000
COVA1-21	0.579	0.384	0.037	0.000
COVA2-31	0.334	0.572	0.095	0.000
COVA1-18	0.249	0.671	0.080	0.000
COVA1-26	0.127	0.710	0.164	0.000
COVA2-02	0.211	0.497	0.292	0.000
COVA1-16	0.112	0.601	0.287	0.000
COVA1-22	0.090	0.522	0.388	0.000
COVA2-07	0.089	0.503	0.408	0.000
COVA1-27	0.182	0.266	0.491	0.062
COVA2-18	0.115	0.127	0.711	0.047
COVA2-15	0.023	0.228	0.628	0.121

Supplemental Table S3. Tabulation of Ab epitopes, neutralization, and $K_{D,app}$

Antibody	Epitope*	Neutralizes?*	$K_{D,app}$ (nM)*
COVA1-25	non-RBD	Yes	N.D.
COVA1-21	non-RBD	Yes	34
COVA2-31	RBD	No	0.3
COVA1-18	RBD	Yes	0.03
COVA1-26	NTD	No	0.9
COVA2-02	RBD	Yes	0.3
COVA1-16	RBD (Up)	Yes	0.3
COVA1-22	NTD	Yes	0.4
COVA2-07	RBD	Yes	0.6
COVA1-27	non-RBD	No	0.7
COVA2-18	S2	No	1.6
COVA2-15	RBD (Up+Down)	Yes	0.6

* Data from ref[12] of main text.

Supplemental Table S4. Tabulation of masses for Ab binding experiments.

Method	Sample	Species	Mass (kDa)	FWHM (kDa)	Normalized Abundance (%)
MP	COVA1-18 + S-trimer (1:1 mix)	S	498	104	69
		S + 1Ab	640	120	31
	COVA1-18 + S-trimer (2:1 mix)	S	501	89	33
		S + 1Ab	662	141	67
	COVA1-18 + S-trimer (3:1 mix)	S	481	92	21
		S + 1Ab	634	118	69
		S + 2Ab	780	101	11
	COVA1-18 + S-trimer (4:1 mix)	S + 1Ab	626	125	81
		S + 2Ab	789	106	19
	COVA2-15 + S-trimer (1:1 mix)	S	482	106	63
		S + 1Ab	634	115	37
	COVA2-15 + S-trimer (2:1 mix)	S	486	92	13
		S + 1Ab	635	111	75
		S + 2Ab	785	120	12
	COVA2-15 + S-trimer (3:1 mix)	S + 1Ab	627	118	16
		S + 2Ab	788	115	73
		S + 3Ab	951	94	11
	COVA2-15 + S-trimer (4:1 mix)	S + 1Ab	654	113	17
		S + 2Ab	800	115	71
		S + 3Ab	956	94	12
CD-MS	COVA1-18 + S-trimer (4:1 mix)	S	480	70	20
		S + 1Ab	627	76	63
		S + 2Ab	771	86	17
	COVA2-15 + S-trimer (4:1 mix)	S	470	65	17
		S + 1Ab	630	77	30
		S + 2Ab	779	87	48
		S + 3Ab	929	91	5
